# Correlation of brain tissue volume loss with inflammatory biomarkers IL1β, P-tau, T-tau, and NLPR3 in the aging cognitively impaired population

**DOI:** 10.3389/fnagi.2024.1388654

**Published:** 2024-07-23

**Authors:** Kyung Mi Lee, Sang Tae Kim, Yunan Tian, Sue Min Jung, Yunjung Chang, Hak Young Rhee, Soonchan Park, Chang-Woo Ryu, Woo-In Lee, Eui Jong Kim, Geon-Ho Jahng

**Affiliations:** ^1^Department of Radiology, Kyung Hee University Hospital, Kyung Hee University College of Medicine, Seoul, Republic of Korea; ^2^JN Pharma, Seongnam-si, Republic of Korea; ^3^Department of Medicine, Graduate School, Kyung Hee University College of Medicine, Seoul, Republic of Korea; ^4^Department of Biomedical Engineering, Undergraduate School, College of Electronics and Information, Kyung Hee University, Yongin-si, Gyeonggi-do, Republic of Korea; ^5^Department of Neurology, Kyung Hee University Hospital at Gangdong, Kyung Hee University College of Medicine, Seoul, Republic of Korea; ^6^Department of Radiology, Kyung Hee University Hospital at Gangdong, Kyung Hee University College of Medicine, Seoul, Republic of Korea; ^7^Department of Laboratory Medicine, Kyung Hee University Hospital at Gangdong, Kyung Hee University College of Medicine, Seoul, Republic of Korea

**Keywords:** inflammasome, blood-based biomarker, interleukin 1-beta, total tau, NLRP3, brain tissue volume

## Abstract

**Background:**

Blood inflammatory biomarkers have emerged as important tools for diagnosing, assessing treatment responses, and predicting neurodegenerative diseases. This study evaluated the associations between blood inflammatory biomarkers and brain tissue volume loss in elderly people.

**Methods:**

This study included 111 participants (age 67.86 ± 8.29 years; 32 men and 79 women). A battery of the following blood inflammatory biomarkers was measured, including interleukin 1-beta (IL1β), NACHT, LRR, and PYD domains-containing protein 3 (NLRP3), monomer Aβ42 (mAβ), oligomeric Aβ42 (oAβ), miR155, neurite outgrowth inhibitor A (nogo-A), phosphorylated tau (P-tau), and total tau (T-tau). Three-dimensional T1-weight images (3D T1WI) of all participants were prospectively obtained and segmented into gray matter and white matter to measure the gray matter volume (GMV), white matter volume (WMV), and gray-white matter boundary tissue volume (gwBTV). The association between blood biomarkers and tissue volumes was assessed using voxel-based and region-of-interest analyses.

**Results:**

GMV and gwBTV significantly decreased as the levels of IL1β and T-tau increased, while no significant association was found between the level of P-tau and the three brain tissue volumes. Three brain tissue volumes were negatively correlated with the levels of IL1β, P-tau, and T-tau in the hippocampus. Specifically, IL1β and T-tau levels showed a distinct negative association with the three brain tissue volume losses in the hippocampus. In addition, gwBTV was negatively associated with the level of NLRP3.

**Conclusion:**

The observed association between brain tissue volume loss and elevated levels of IL1β and T-tau suggests that these biomarkers in the blood may serve as potential biomarkers of cognitive impairment in elderly people. Thus, IL1β and T-tau could be used to assess disease severity and monitor treatment response after diagnosis in elderly people who are at risk of cognitive decline.

## Introduction

Structure changes at the gray matter (GM) level are associated with cognitive decline and memory dysfunction during aging, primarily due to neuronal death, degeneration of the neuropil, and de-arborization of the dendritic network (Freeman et al., 2008; Alegret et al., 2010; Fjell et al., 2014). Moreover, white matter (WM) integrity contributes to age-related cognitive decline, which is primarily mediated by axonal damage, myelin loss, and myelin degeneration (Gunning-Dixon and Raz, 2000; Lebel et al., 2012; Preziosa et al., 2023). Brain atrophy is often observed in patients with Alzheimer's disease (AD) and amnestic mild cognitive impairment (MCI) in the medial temporal lobe (Leow et al., 2009; Jack et al., 2011). Gray matter volume (GMV) has been found to be negatively correlated with age (Fotenos et al., 2005; Smith et al., 2007; Taki et al., 2011). However, studies focusing on the association between certain blood-based biomarkers and the loss of brain tissue volume are insufficient.

The immune system undergoes an age-related process characterized by increased proinflammatory cytokine production, contributing to cognitive decline (Franceschi et al., 2000, 2007). The interplay between aging and neuroinflammation involves a complex network of cytokines, signaling pathways, and metabolic reactions, significantly influencing neuronal survival, proliferation, differentiation, and plasticity (Rea et al., 2018). In the aging brain, diminished microglial homeostatic functions lead to the accumulation of toxic compounds, thereby exacerbating cognitive decline, particularly in the context of neurodegenerative diseases (Hagemeyer et al., 2017; d'Avila et al., 2018). Microglia, the primary resident immune cells of the central nervous system (CNS), play a pivotal role in maintaining brain homeostasis. Dysfunction in microglia triggers a cascade of events that leads to neuroinflammation, synaptic disruption, and compromised neuronal signaling—key factors contributing to cognitive impairment (Zhang et al., 2021; Zhuo et al., 2023). Within the domain of cognitive dysfunction, the mechanical and signaling impacts of microglial dysfunction are profound. Pathological changes such as neuroinflammation (Singhal and Baune, 2017), impaired synaptic pruning (Miao et al., 2023), and phagocytic dysfunction (Gao et al., 2023) are some of the core issues stemming from microglial malfunction. Further, dysfunctional microglia release proinflammatory cytokines and free radicals, creating a neurotoxic environment detrimental to cognitive functions (Muzio et al., 2021; Zhuo et al., 2023). An imbalance in synaptic pruning by microglia disrupts neural circuits and impairs cognitive abilities (Rim et al., 2024), while deficient phagocytic activity leads to the buildup of misfolded proteins and cellular debris, exacerbating neurodegenerative conditions (Gao et al., 2023). Moreover, signaling pathways central to microglial function are compromised, impacting crucial cognitive processes such as microglia-neuron communication, the management of protein aggregates integral to Alzheimer's disease, and the regulation of reactive oxygen species (ROS) (Miao et al., 2023). Disruption in microglial-neuronal communication can significantly affect learning and memory (Singhal and Baune, 2017). Additionally, dysfunctional microglia may facilitate the spread of pathological protein aggregates, like tau and amyloid beta, linked with cognitive decline in diseases such as Alzheimer's disease (Gao et al., 2023). Overactivation of microglia can also result in excessive ROS production, damaging neurons and synapses, and further impairing cognitive function (Gao et al., 2023). Given these challenges, targeting inflammatory cytokines has become a crucial area of research in treating and managing neurodegenerative diseases (Zhang et al., 2023). Consequently, monitoring inflammatory factors has become essential to contemporary neurodegenerative disease management strategies.

Prior research has identified various blood markers for AD, with investigations focusing on amyloid-beta (Aβ) (Olsson et al., 2016; Nakamura et al., 2018), tau proteins (Olsson et al., 2016; Mielke et al., 2018), and neurofilament light—a structural protein indicating neurodegeneration (Mattsson et al., 2017; Khalil et al., 2018). Aβ42, a key neurotoxic form linked to Alzheimer's pathology, is generated from amyloid precursor proteins and eventually forms plaques (Kurucu et al., 2022). Furthermore, microRNAs (miRs), such as miR155, are under study as potential biomarkers for their role in immune response regulation, inflammation control in the brain, and their stable presence in blood transport systems. In particular, Aβ can activate the NLRP3 inflammasome in microglia, leading to increased interleukin-1 beta (IL1β) production, exacerbating neuroinflammation, and advancing Alzheimer's progression. This study focuses on these molecules as potential blood-based biomarkers for AD.

MicroRNAs (miRs) are crucial for regulating gene expression, directly impacting protein synthesis. Due to their regulatory functions, miRs are being explored as potential biomarkers for diagnosing and monitoring AD progression. Notably, miRs in AD are implicated in several aspects of the disease's pathogenesis, including the regulation of amyloid precursor protein (APP) (Wei et al., 2020) and tau protein pathways (Abuelezz et al., 2021), neuroinflammation (Lukiw, 2023), and neurodegeneration (Arora et al., 2022). Certain miRs can bind to the messenger RNA (mRNA) of APP, the precursor to Aβ peptides that form plaques in AD-affected brains. By influencing APP levels, miRs can affect Aβ production and plaque formation (Femminella et al., 2015). Similarly, miRs target the mRNA of tau proteins, which is associated with the formation of neurofibrillary tangles in AD. Disruptions in miR regulation can lead to the hyperphosphorylation and aggregation of tau proteins (Wei et al., 2020). Additionally, specific miRs can regulate immune responses in the brain by targeting mRNAs of inflammatory mediators. Alterations in miR levels can influence microglial activation and neuroinflammation, key features of AD pathology (Wei et al., 2020; Abuelezz et al., 2021). miRs also play a role in targeting mRNAs related to cell survival and apoptosis, thereby affecting neuronal survival. Dysregulated miR expression can increase neuronal death and neurodegeneration (Abuelezz et al., 2021). Furthermore, the viability of miRs as biomarkers is supported by their transportation within liposomes, high-density lipoproteins, exosomes, and other protective proteins, safeguarding them from degradation (Leidinger et al., 2013; Zendjabil, 2018; Nagaraj et al., 2019). Proper regulation of various T cells, influenced by miR155, can mitigate AD-related pathologies (Song and Lee, 2015).

The primary signal of cerebral neuroinflammation for IL1β expression and subsequent inflammatory events in microglia can be driven by Aβ activation of the NACHT, LRR, and PYD domains-containing protein 3 (NLRP3) inflammasome in microglia (Lučiunaite et al., 2020). Elevation in IL1β levels and the resulting neuroinflammatory processes and leukocyte recruitment to CNS play a detrimental role in AD progression. IL1β has been shown to increase the expression of APP in neuronal cultures (Kim and Bezprozvanny, 2023) and promote neuronal tau phosphorylation and tangle formation in the brain (Wang et al., 2023). The neurite outgrowth inhibitor A (nogo-A), a potent myelin-associated inhibitor, is predominantly expressed in oligodendrocytes of the CNS. The extracellular surface expresses its axon growth inhibiting domain of 66 amino acids (Nogo-66) (Dave et al., 2023; Pavon et al., 2023). Consequently, monomer Aβ42 (mAβ), oligomeric Aβ42 (oAβ), NLRP3, IL1β, miR155, nogo-A, phosphorylated tau (P-tau), and total tau (T-tau) were the targeted blood-based biomarkers used in this study.

The contribution of inflammatory factors to the loss of brain tissue in cognitively impaired elderly people is still unknown. Plasma levels of blood biomarkers might be associated with brain tissue volume loss in cognitively impaired people as they age. This study evaluated the associations between blood inflammatory biomarkers and brain tissue volume loss in elderly participants with cognitive impairment.

## Materials and methods

### Standard protocol approval, registration, and patient consent

This prospective study was conducted in compliance with the Declaration of Helsinki and received approval from the Institutional Review Board (IRB) of Kyung Hee University Hospital at Gangdong (KHNMC IRB 2009-056, KHNMC GRRB 2009-004, KHNMC IRB 2011-059, KHNMC GRRB 2011-008) for studies involving human subjects. All participants provided informed consent.

### Study participants

We included 111 participants with ages ranging from 49 to 87 years (mean age 67.86 ± 8.29 years) in this study. Participants were prospectively recruited by the neurological center at our institution and underwent neurological examination, standard neuropsychological testing (Ahn et al., 2010), and MRI scans. Cognitive functions were assessed using the full version of the Seoul Neuropsychological Screening Battery (SNSB) (Ahn et al., 2010), and global cognitive ability was evaluated using the Korean version of the Mini-Mental State Examination (K-MMSE) and the Clinical Dementia Rating (CDR). Only participants with MRI data and blood samples were included in the study.

Participants included both cognitively normal and impaired elderly individuals. Cognitive impairment evaluations were based on Petersen's criteria (Petersen et al., 1999, 2001) and the criteria of the National Institute of Neurological and Communicative Disorders and Stroke-Alzheimer Disease and Related Disorders Association (NINCDS-ADRDA) (McKhann et al., 1984). Participants' cognition met Petersen's criteria for impairment and the NINCDS-ADRDA criteria for dementia, as applicable. Cognitively normal participants were healthy volunteers without a medical history of neurological disease who had a normal brain MRI. Clinical Dementia Rating (CDR) scores included in this study were 0, 0.5, 1, or 2. Brain MRI screenings confirmed the absence of structural lesions, such as cerebral hemorrhage or infarction, hippocampal sclerosis, brain tumors, traumatic encephalomalacia, and vascular malformation. The exclusion criteria included a history of psychological disease, stroke, brain surgery, seizure, head trauma, severe cerebral white matter hyperintensities defined by the modified Fazekas scale (Seo et al., 2009), or current systemic medical conditions that could affect cognition. We excluded patients with severe cognitive impairment (CDR > 2) and those with vascular or frontotemporal dementia due to their distinct brain atrophy patterns and potential difficulties in obtaining high-quality brain MRI images.

### Laboratory tests

After obtaining consent from the patient, blood was collected in a 3-ml ethylenediamine tetraacetic acid (EDTA) tube. EDTA blood remained as whole blood. Serum was separated from serum separator tube (SST) blood, and 1 ml of each was dispensed and stored at −70°C. This study did not include genetic information, such as apolipoprotein E (APOE) genotyping.

### Quantification of plasma levels

#### Preparation of primary antibodies

EDTA (ethylenediaminetetraacetic acid), 1-Ethyl-3-(3-dimethylaminopropyl)-carbodiimide (EDC), and N-hydroxysuccinimide (NHS) were obtained from Sigma-Aldrich (Sigma-Aldrich, Merck, USA). The following primary antibodies were used: nogo-A (ab62024, Cambridge), oAβ (AB9234, Chemicon International), and NLRP3 (NBP2-12446, Novus Biologicals). Other primary antibodies used for ICC (immunocytochemistry analysis) were mAβ (sc-32277, Santa Cruz), T-tau (sc-32274, Santa Cruz), P-tau (sc-32275, Santa Cruz), and IL1β (sc-32294, Santa Cruz). The following ELISA kits were used: Human Nogo-A ELISA Kit (MBS1608944, MyBioSource), oAβ (27725, IBL-America (Immuno-Biological Laboratories), NLRP3 (LS-F17336, LSBio), mAβ (OKEH00815, Aviva Systems Biology), T-tau (LS-F6386, LSBio), P-tau (OKAG02072, Aviva Systems Biology), miRNA155 (RDM0017H, R&D), and IL1β (ELH-IL1b-5, RayBio).

Primary antibodies were conjugated with QD565 (1:5, Molecular Probes) (Eugene, USA). QD525/565 was purchased from Molecular Probes (Thermo Fisher Scientific Inc., MA, USA). We purchased 1-(3-(dimethylamino) propyl)-3-ethylcarbodiimide hydrochloride (EDC) and N-hydroxylsuccinimide (NHS) from Acros Organics (Geel, Belgium). A monoclonal antihuman IL1β antibody was obtained from Sigma-Aldrich (St. Louis, MO, USA). All antibodies were diluted in phosphate-buffered saline (PBS). Typically, 4 mg of EDC and 2 mg of NHS were added to the antibody-QD565 solution to provide NHS-activated antibody after 1 h of reaction. After drying, the NHS-activated antibody was reacted with QD525 in 2 mL of Tris-EDTA solution (0.1 M NaCl, pH 8.0) for 1 h at an antibody/QD525 molar ratio of 1:5. Subsequently, the final solution was extensively dialyzed and lyophilized overnight in a freeze-dryer to obtain the final product.

#### Molecular beacon imaging for miR155

A molecular beacon probe for miR155 imaging was designed with an oligonucleotide linked to a stem sequence that functioned as a switch at the 5′-end of the oligonucleotide (Bioneer, Korea). QD565-carboxyl groups were purchased from Molecular Probes (Thermo Fisher Scientific, Waltham, MA, USA). To construct miR155-QD565 MB, an amine-modified molecular beacon, miR155 MB (5′-cgccaatggggatagtgctaatcgtaattttca-3′), was purchased from Bioneer Inc. (Daejeon, Korea). Following a previous report, the miR155 MB was designed as a partly double-stranded sequence (Hwang et al., 2010). In addition, the linker region had a stem sequence (CAGCG), which was complementary to a DNA sequence for molecular imaging. A single on/off molecule was conjugated to BHQ2 (Black Hole Quencher 2) at the 3′-end of the oligonucleotide region, serving as a quencher (Bioneer, Korea).

To prepare the QD565 miRNA-155 MB for an *in vitro* experiment, we designed a single-stranded oligonucleotide with amine parts at the cohesive end. Then, we linked it with a beacon linker sequence. According to the manufacturer's method, the NH2-miRNA-155 sequence was conjugated with the COOH-QD565 probe (designated as fluorophore dye) at a molar ratio of 2:1 in 0.1 M TE buffer (pH 7.0) with 78 mg/mL EDC (1-ethyl-3-(3-dimethylaminopropyl)carbodiimide hydrochloride) for 2 h at room temperature. To form a secondary structure for the high binding ability of the Mir-155 MB sequence at the miR-155 target binding active site on the cell surface, 5 pmol of QD_565_ miR-155 MB was thermodynamically annealed at 94 and 72°C in a PCR condition to construct a blood-based miR-155 sequence.

#### Analysis of fluorescence spectra

First, 10 μL of plasma was mixed with 10 μL of miR155 MB probe with 80 μL of distilled water to reach a final volume of 100 μL. The spectral wavelength range was set to 565–625 nm, with a fixed slit size and photomultiplier tube gain. For the plasma samples, the background signal was determined by measuring the fluorescence intensity of a mixture containing the miRNA MB without fluorescence labeling. The (*F*-*F0*)/*F0* value was calculated to determine the concentration of miRNAs using the acquired regression equation. In this calculation, *F0* represented the fluorescence intensity at 595 nm without the template miRNAs, and *F* represented the fluorescence intensity with the plasma-derived miRNA, with the background subtracted.

#### Confocal microscopy analysis

Plasma was placed on a glass slide and maintained for 5 min. Each antibody-QD525 probe was then reacted with the plasma, and a cover glass was applied immediately after the 5-min reaction. For miRNA155 imaging, the 5′-end of the MB containing the miR155-binding sequence was conjugated with QD565 (absorbance/emission wavelength: 565/625 nm). Plasma images were captured using confocal microscopy (LSM710, Carl Zeiss, Jena, Germany). This method enabled accurate and simultaneous *in vitro* detection of miRNA155 and each antibody-QD525 level in the plasma.

### MRI acquisition and imaging processing

#### MRI acquisition

A sagittal structural three-dimensional (3D) T1-weighted (T1W) image was captured to evaluate the relationship between blood biomarkers and brain tissue volumes using a turbo field echo sequence with the following parameters: repetition time (TR) = 8.1 ms, echo time (TE) = 3.7 ms, flip angle (FA) = 8°, field-of-view (FOV) = 236 × 236 mm^2^, and voxel size =1 × 1 × 1 mm^3^. In addition, T2-weighted turbo-spin-echo and fluid-attenuated inversion recovery images were acquired to examine any brain malformations. MRI images were acquired for each subject using a clinical 3T MRI system (Achieva, Philips Medical Systems, Best, the Netherlands).

#### Image processing

The 3D T1W image was segmented into GM, WM, and CSF and spatially normalized to our dementia template (Guo et al., 2021) using the computational anatomy toolbox (CAT12) segmentation tool (Seiger et al., 2018) to obtain GMV and WMV. In addition, to obtain the gray and white matter boundary tissue volume (gwBTV), information on the segmented GM and WM brain tissue locations was applied to the normalized 3D T1W image to threshold the signal intensity to create a binary map (Tian et al., 2023). The threshold of the binary image was mainly defined by the mean and standard deviation (SD) over the partial volume percentage of GM and WM as 1 and 0, respectively. Subsequently, the binary image was converted to calculate the gwBTV map for each participant. Finally, the brain tissue volumes of GMV, WMV, and gwBTV maps for each participant were smoothed using a Gaussian kernel of 8 × 8 × 8 mm^3^ full width at half maximum (FWHM) for the voxel-based statistical analyses. Statistical Parametric Mapping Version 12 (SPM12) software (Wellcome Department of Imaging Neuroscience, University College, London, UK) was used for the image post-processing.

### Statistical analyses

#### Demographic characteristics, results of neuropsychological tests, and blood biomarkers

The correlation between the variables of the participant's demographic characteristics, such as age, years of education, and K-MMSE scores, and the levels of each blood biomarker were analyzed using Pearson correlation analysis.

#### Voxel-based multiple regression analyses between brain tissue volumes and plasma biomarkers

The relationship between brain tissue volume loss and the level of each blood biomarker in men, women, and all participants was assessed using voxel-based multiple regression analysis for GMV, WMV, and gwBTV separately. In this analysis, the dependent variable was the voxel value of each brain tissue volume, while the independent variable was the level of each blood biomarker within the framework of a general linear model adjusted for the participant's age and years of education. The negative or positive association of each brain tissue volume with the levels of each plasma biomarker was evaluated. A significance level of α = 0.05 was applied, with correction for multiple comparisons using a false-discovery rate (FDR) and a minimum cluster size of at least 100 contiguous voxels.

#### Region-of-interest-based correlation analyses

For ROI-based analysis of the three brain tissue volumes, atlas-based ROIs were defined for the bilateral hippocampi, parahippocampal gyrus, precuneus, middle temporal gyrus, middle frontal gyrus, and middle occipital gyrus in the brain using wfu_pickAtlas software (https://www.nitrc.org/projects/wfu_pickatlas/). These main areas were selected based on the results of voxel-based multiple regression analyses. In addition, we selected other brain areas that are related to cognitive function, such as the anterior cingulate, brain stem, cerebellum, pons, striatum, caudate nucleus, entorhinal cortex, thalamus, inferior frontal gyrus, superior frontal gyrus, inferior temporal gyrus, and superior temporal gyrus. GMV, WMV, or gwBTV values for each ROI were extracted from all participants using Marsbar software (Matthew Brett, http://marsbar.sourceforge.net). The following analyses were performed: First, a Pearson correlation heatmap analysis was conducted to investigate the relationship between the values of the three brain tissue volumes and the levels of blood biomarkers for each ROI. Second, a simple regression analysis evaluated the associations between GMV, WMV, or gwBTV and all blood biomarkers for each ROI. The initial model was “ROI tissue volume = β1age+ β2education+ β3^*^each blood biomarker + error.” Each brain tissue volume in each ROI was described as a dependent variable, and all blood biomarkers were described as independent variables. A variable was entered into the model if the *p*-value of each dependent variable was < 0.05 and was removed from the model if the *p*-value was >0.1. The Medcalc statistical program (MedCalc Software, Acacialaan, Ostend, Belgium) was used for all statistical analyses except for the heatmap analysis, which was conducted using R software.

## Results

### Participant characteristics

Demographic data, neuropsychological test scores, and plasma biomarker levels are shown in Table 1. The average age of the participants was 67.86 years (range: 49–87), with a gender distribution of 32 men and 79 women. The average K-MMSE score was 24.30 (range: 11–30), and the average educational attainment was 8.28 years (range: 0–20).

**Table 1:** Summary of demographic data, results of neuropsychologic tests, blood-based biomarkers targets for participants, and results of the correlation of blood biomarkers with age, K-MMSE scores, and the year of education.

**Demographic data and neuropsychologic tests**				
No. of subjects	111			
^*^Age(years)	67.86 ± 8.29 (49.00–87.00)			
Sex (male/female)	32/79			
^*^K-MMSE score (/30)	24.30 ± 5.64 (11.00–30.00)			
CDR (range)	1 (0–2)			
^*^Education(years)	8.28 ± 5.21 (0.00–20.00)			
^*^ **Plasma levels of the blood biomarkers**		^#^ **Correlations (** ***r*** **/** ***p*** **)**		
		**Ages**	**K-MMSE scores**	**Education-years**
mAβ42 (pg/ml)	10.73 ± 6.69 (0.43–29.88)	*r* = 0.102, *p* = 0.289	*r* = −0.179, *p* = 0.060	*r* = −0.148, *p* = 0.122
oAβ42 (pg/ml)	3.80 ± 2.15 (0.50–8.80)	*r* = 0.093, *p* = 0.333	*r* = −0.322, *p* < 0.001	*r* = −0.104, *p* = 0.277
NLRP3 (ng/ml)	29.98 ± 18.45 (1.50–106.19)	*r* = −0.063, *p* = 0.510	*r* = 0.045, *p* = 0.641	*r* = 0.164, *p* = 0.086
IL1β (pg/ml)	24.90 ± 12.81 (4.40–48.00)	*r* = 0.240, *p* = 0.011	*r* = −0.608, *p* < 0.001	*r* = −0.182, *p* = 0.056
miR155 (fg/ml)	145.41 ± 89.63 (10.89–415.91)	*r* = 0.206, *p* = 0.030	*r* = −0.271, *p* = 0.004	*r* = −0.043, *p* = 0.657
nogo-A (fg/ml)	4.12 ± 2.97 (0.28–15.62)	*r* = 0.132, *p* = 0.168	*r* = −0.316, *p* < 0.001	*r* = 0.057, *p* = 0.554
P-tau (pg/ml)	12.26 ± 7.79 (0.90–32.60)	*r* = 0.246, *p* = 0.009	*r* = −0.687, *p* < 0.001	*r* = −0.281, *p* = 0.003
T-tau (pg/ml)	7.41 ± 5.07 (1.09–18.7)	*r* = 0.263, *p* = 0.005	*r* = −0.689, *p* < 0.001	*r* = −0.245, *p* = 0.010

^*^The continuous variables in the demographic data and neuropsychologic tests and plasma levels of the blood biomarkers are listed as mean ± standard deviation (range), but the CDR scores are presented as the median (range) value.

^#^The results of the Pearson correlation analysis for all blood biomarkers with the variables of the participant's demographic characteristics are listed as the correlation coefficient (*r*) and *p*-value.

K-MMSE, the Korean version of the Mini-Mental State Examination; CDR, Clinical Dementia Rating; mAβ, monomer amyloid-beta; oAβ, oligomeric amyloid-beta; IL1β, interleukin 1-beta; miR155, microRNA-155; P-tau, phosphorylated tau; and T-tau, total tau.

As disease severity increased, so did the plasma levels of specific biomarkers. Table 1 presents these levels for all participants: mAβ at 10.73 pg/ml, oAβ at 3.80 pg/ml, NLRP3 at 29.98 ng/ml, IL1β at 24.90 pg/ml, miR155 at 145.41 fg/ml, nogo-A at 4.12 fg/ml, P-tau at 12.26 pg/ml, and T-tau at 7.41 pg/ml.

Correlations between biomarkers and age, K-MMSE scores, and education levels are also detailed in Table 1. Age showed a positive correlation with IL1β, miR155, P-tau, and T-tau levels. Conversely, K-MMSE scores were inversely correlated with oAβ, IL1β, nogo-A, P-tau, and T-tau levels. Education was negatively associated with P-tau and T-tau levels.

### Voxel-based multiple regression analysis

**Figure 1:**
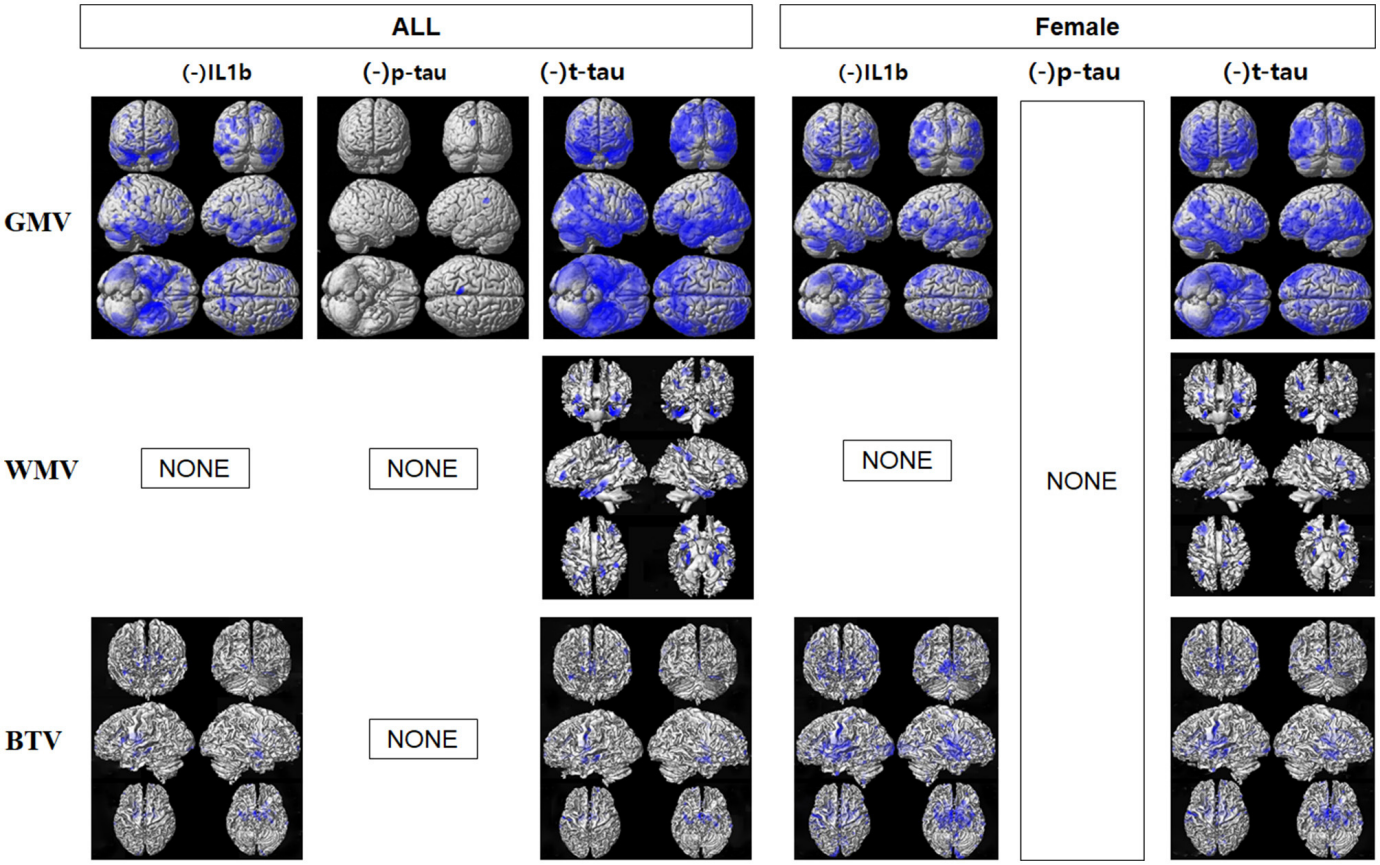
Results of voxel-based multiple regression analyses between gray matter volume (GMV), white matter volume (WMV), or gray-white matter boundary tissue volume (gwBTV) of female and all participants and levels of blood-based biomarkers after adjusting for participants' age and education years. The blue color indicates the negative relationship between brain tissue volumes and levels of IL1β, P-tau, and T-tau. There were no positive relationships between brain tissue volumes and levels of any blood-based biomarkers. There were no significant correlations between brain tissue volumes and levels of monomer Aβ (mAβ), NLRP3, miR155, oligomer Aβ (oAβ), and Nogo-A. There was no correlation between brain tissue volumes and P-tau in the female participants. There was no correlation between brain tissue volumes and blood biomarkers in the male participants.

The voxel-based multiple regression analysis demonstrated a negative correlation between the levels of IL1β, P-tau, and T-tau and GMV across all participants, with significant findings, particularly in the temporal, occipital, parietal, and frontal regions, as shown in Figure 1 and detailed in Supplementary Table S1. Additionally, T-tau levels were negatively associated with WMV in the parahippocampal gyrus, precuneus, and middle temporal gyrus, as recorded in Supplementary Table S2. Both IL1β and T-tau were inversely correlated with gwBTV across multiple regions, including the frontal, temporal, occipital, and parietal lobes, as detailed in Supplementary Table S3. The regression analysis did not reveal significant correlations between brain tissue volumes and the levels of other blood biomarkers under study.

In female participants, IL1β levels were negatively correlated with both GMV and gwBTV, while T-tau levels were inversely related to all three measured brain tissue volumes. Notably, there was no significant correlation between P-tau and brain tissue volumes in female participants. Additionally, the analysis did not show any correlation between brain tissue volumes and blood biomarkers in male participants, which could be attributed to the limited sample size of male participants in this study.

### ROI-based analysis

#### Heatmap analysis of Pearson correlations

**Figure 2:**
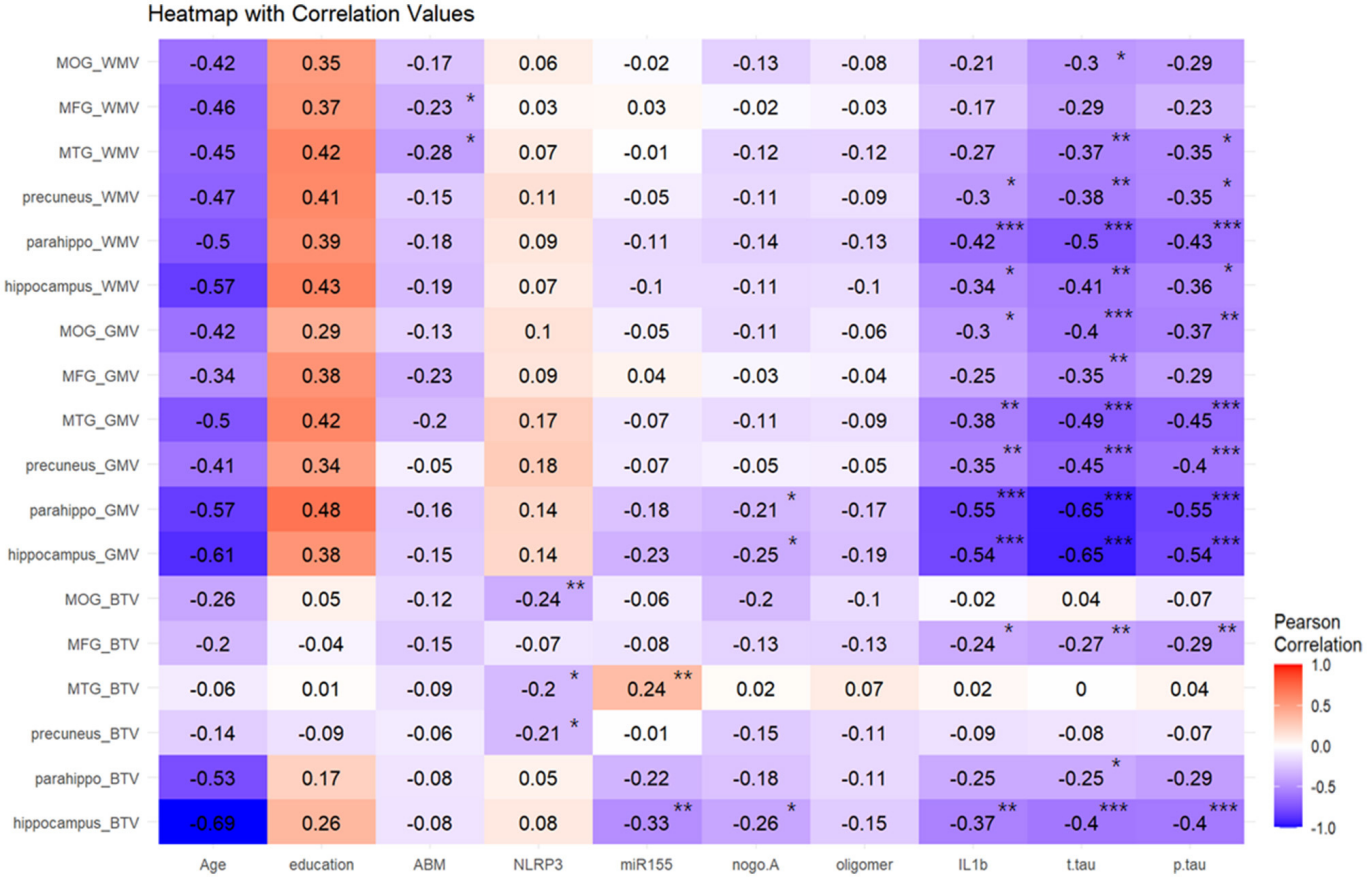
Result of the Pearson correlation heatmap analysis between blood biomarkers and brain tissue volumes in the specific brain area. Blood biomarkers signed with * were found to be significant at a *p*-value of < 0.05, blood biomarkers signed with ** were found to be significant at a *p*-value of < 0.01, and blood biomarkers signed with *** were found to be significant at *p* < 0.001. Color coding indicates the strength of the correlation between all blood biomarkers and brain tissue volumes. WMV, white matter volume; GMV, gray matter volume; gwBTV, gray-white matter boundary tissue volume; MOG, middle occipital gyrus; MFG, middle frontal gyrus; MTG, middle temporal gyrus; Parahippo, parahippocampal gyrus; ABM(mAβ), monomer amyloid-beta; NLRP3, NACHT, LRR, and PYD domains-containing protein 3; miR155, microRNA-155; nogo-A, neurite outgrowth inhibitor A; oligomer (oAβ), oligomeric amyloid-beta; IL1β, interleukin 1-beta; T-tau, total tau; P-tau, phosphorylated tau.

Figure 2 presents a heatmap derived from the Pearson correlation analysis that evaluated the relationships between various blood biomarkers and tissue volumes across different brain regions. Age generally exhibited a negative correlation with all three tissue volumes, whereas education level displayed a positive correlation. Specifically, WMV in the middle frontal and middle temporal gyri was negatively correlated with mAβ. gwBTV in the middle occipital gyrus, middle temporal gyrus, and precuneus showed negative correlations with NLRP3. Additionally, gwBTV in the middle temporal gyrus positively correlated with miR155 yet displayed a negative correlation in the hippocampus. Both GMV and gwBTV in the parahippocampal gyrus and hippocampus were negatively correlated with nogo-A. All three tissue volumes—GMV, WMV, and gwBTV—exhibited significant negative correlations with IL1β, T-tau, and P-tau. Except for the middle frontal gyrus, most ROIs showed significant negative correlations with GMV due to these biomarkers. WMV in the middle temporal gyrus, precuneus, parahippocampal gyrus, and hippocampus negatively correlated with IL1β, T-tau, and P-tau. Notably, gwBTV in the hippocampus and middle frontal gyrus was also negatively associated with these biomarkers. No significant correlations were noted between oAβ and the three tissue volumes.

Supplementary Table S4 summarizes the results of the correlation analysis between other brain areas and biomarkers. In regions specifically associated with cognitive function, such as the anterior cingulate and entorhinal cortex, GMV, WMV, and gwBTV were negatively correlated with the levels of IL1β, P-tau, and T-tau, indicating extensive associations across cognitive-related brain structures.

#### Regression analysis

Table 2 presents the regression analysis assessing the relationship between brain tissue volumes (GMV, WMV, and gwBTV) and blood biomarkers across different ROIs. T-tau levels showed the most robust negative association with all three tissue volumes, particularly in the hippocampus. This association was observed in GMV and WMV across all ROIs, except the middle occipital gyrus. P-tau levels were negatively correlated with GMV in all ROIs, barring the middle frontal gyrus. WMV showed a similar negative correlation in most ROIs, except for the middle frontal and middle occipital gyri. Additionally, gwBTV in the hippocampus, parahippocampal, and middle frontal gyrus also exhibited negative associations with P-tau.

**Table 2:** The results of a simple regression analysis of brain tissue volumes with blood biomarkers in specific brain areas.

**Index**	**Tissue volume**	**mAβ**	**Oligomer**	**IL1β**	**miR155**	**NLRP3**	**Nogo A**	**P-tau**	**T-tau**
Hippocampus	GMV	β = −0.070	β = −0.119	β = −0.408	β = −0.115	β = 0.080	β = −0.189	β = −0.397	β = −0.510
		*P* = 0.358	*P* = 0.112	*P* < 0.001	*P* = 0.132	*P* = 0.290	*P* = 0.012	*P* < 0.001	*P* < 0.001
	WMV	β = −0.111	β = −0.030	β = −0.195	β = 0.005	β = −0.0004	β = −0.058	β = −0.192	β = −0.243
		*P* = 0.150	*P* = 0.697	*P* = 0.012	*P* = 0.951	*P* = 0.996	*P* = 0.455	*P* = 0.016	*P* = 0.002
	gwBTV	β = −0.013	β = −0.089	β = −0.223	β = −0.202	β = 0.038	β = −0.179	β = −0.253	β = −0.245
		*P* = 0.855	*P* = 0.209	*P* = 0.002	*P* = 0.004	*P* = 0.593	*P* = 0.011	*P* < 0.001	*P* = 0.001
Parahippo	GMV	β = −0.069	β = −0.101	β = −0.408	β = −0.074	β = 0.068	β = −0.173	β = −0.394	β = −0.497
		*P* = 0.361	*P* = 0.178	*P* < 0.001	*P* = 0.329	*P* = 0.370	*P* = 0.020	*P* < 0.001	*P* < 0.001
	WMV	β = −0.108	β = −0.071	β = −0.302	β = −0.014	β = 0.023	β = −0.097	β = −0.288	β = −0.365
		*P* = 0.188	*P* = 0.385	*P* < 0.001	*P* = 0.867	*P* = 0.776	*P* = 0.237	*P* = 0.001	*P* < 0.001
	gwBTV	β = −0.030	β = −0.068	β = −0.137	β = −0.117	β = 0.021	β = −0.108	β = −0.182	β = −0.131
		*P* = 0.719	*P* = 0.414	*P* = 0.106	*P* = 0.164	*P* = 0.802	*P* = 0.193	*P* = 0.034	*P* = 0.129
Precuneus	GMV	β = 0.019	β = 0.001	β = −0.248	β = 0.010	β = 0.133	β = −0.016	β = −0.294	β = −0.342
		*P* = 0.832	*P* = 0.993	*P* = 0.005	*P* = 0.912	*P* = 0.127	*P* = 0.853	*P* < 0.001	*P* < 0.001
	WMV	β = −0.077	β = −0.030	β = −0.174	β = 0.036	β = 0.044	β = −0.080	β = −0.204	β = −0.245
		*P* = 0.354	*P* = 0.718	*P* = 0.039	*P* = 0.667	*P* = 0.593	*P* = 0.334	*P* = 0.017	*P* = 0.004
	gwBTV	β = −0.065	β = −0.112	β = −0.078	β = 0.029	β = −0.197	β = −0.119	β = −0.078	β = −0.070
		*P* = 0.501	*P* = 0.238	*P* = 0.428	*P* = 0.762	*P* = 0.038	*P* = 0.213	*P* = 0.437	*P* = 0.479
Mid TG	GMV	β = −0.122	β = −0.025	β = −0.248	β = 0.023	β = 0.106	β = −0.070	β = −0.303	β = −0.347
		*P* = 0.132	*P* = 0.755	*P* = 0.002	*P* = 0.778	*P* = 0.189	*P* = 0.387	*P* < 0.001	*P* < 0.001
	WMV	β = −0.210	β = −0.061	β = −0.145	β = 0.080	β = 0.004	β = −0.098	β = −0.202	β = −0.227
		*P* = 0.011	*P* = 0.462	*P* = 0.086	*P* = 0.344	*P* = 0.977	*P* = 0.239	*P* = 0.019	*P* = 0.008
	gwBTV	β = −0.083	β = 0.076	β = 0.040	β = 0.260	β = −0.211	β = 0.028	β = 0.055	β = 0.017
		*P* = 0.394	*P* = 0.435	*P* = 0.690	*P* = 0.008	*P* = 0.030	*P* = 0.775	*P* = 0.591	*P* = 0.864
Mid FG	GMV	β = −0.169	β = 0.008	β = −0.157	β = 0.107	β = 0.032	β = −0.017	β = −0.170	β = −0.238
		*P* = 0.053	*P* = 0.925	*P* = 0.081	*P* = 0.231	*P* = 0.717	*P* = 0.846	*P* = 0.063	*P* = 0.008
	WMV	β = −0.167	β = 0.025	β = −0.043	β = 0.118	β = −0.033	β = 0.019	β = −0.077	β = −0.146
		*P* = 0.047	*P* = 0.766	*P* = 0.616	*P* = 0.167	*P* = 0.695	*P* = 0.826	*P* = 0.382	*P* = 0.093
	gwBTV	β = −0.148	β = −0.125	β = −0.222	β = −0.035	β = −0.066	β = −0.092	β = −0.292	β = −0.257
		*P* = 0.118	*P* = 0.186	*P* = 0.021	*P* = 0.720	*P* = 0.487	*P* = 0.333	*P* = 0.003	*P* = 0.008
Mid OG	GMV	β = −0.077	β = −0.014	β = −0.194	β = 0.030	β = 0.059	β = −0.073	β = −0.267	β = −0.293
		*P* = 0.379	*P* = 0.088	*P* = 0.029	*P* = 0.738	*P* = 0.506	*P* = 0.406	*P* = 0.003	*P* = 0.001
	WMV	β = −0.108	β = −0.025	β = −0.092	β = 0.066	β = 0.002	β = −0.107	β = −0.160	β = −0.177
		*P* = 0.211	*P* = 0.776	*P* = 0.299	*P* = 0.449	*P* = 0.981	*P* = 0.218	*P* = 0.074	*P* = 0.046
	gwBTV	β = −0.101	β = −0.084	β = 0.043	β = −0.005	β = −0.257	β = −0.164	β = −0.018	β = 0.106
		*P* = 0.285	*P* = 0.368	*P* = 0.656	*P* = 0.954	*P* = 0.006	*P* = 0.082	*P* = 0.854	*P* = 0.278

The results of simple regression for all blood biomarkers are listed as the correlation coefficient (β) and *p*-value. The initial model was ROI tissue volume = β1^*^age+ β2^*^education+ β3^*^each blood biomarker + error.

GMV, gray matter volume; WMV, white matter volume; gwBTV, gray-white matter boundary tissue volume; Parahippo, parahippocampal gyrus; Mid TG, middle temporal gyrus; Mid FG, middle frontal gyrus; Mid OG, middle occipital gyrus; mAβ, monomer amyloid-beta; oAβ, oligomeric amyloid-beta; IL1β, interleukin 1-beta; miR155, microRNA-155; P-tau, phosphorylated tau; and T-tau, total tau.

IL1β levels were negatively correlated with GMV in all ROIs except the middle frontal gyrus. WMV in the hippocampus, parahippocampal, precuneus, and gwBTV in the hippocampus and middle frontal gyrus were negatively associated with IL1β. mAβ levels were negatively correlated with WMV in the middle temporal gyrus (β = −0.210, *P* = 0.011) and the middle frontal gyrus (β = −0.167, *P* = 0.047). miR155 levels showed a positive correlation with gwBTV in the middle temporal gyrus (β = −0.202, *P* = 0.004) but a negative correlation with gwBTV in the hippocampus (β = 0.260, *P* = 0.008). NLRP3 levels were negatively associated with gwBTV in the precuneus (β = −0.197, *P* = 0.038), middle temporal gyrus (β = −0.211, *P* = 0.030), and middle occipital gyrus (β = −0.257, *P* = 0.006). Nogo-A was negatively correlated with GMV and gwBTV in the hippocampus and parahippocampal regions. No significant associations were found between the three brain tissue volumes and oAβ levels.

## Discussion

**Figure 3:**
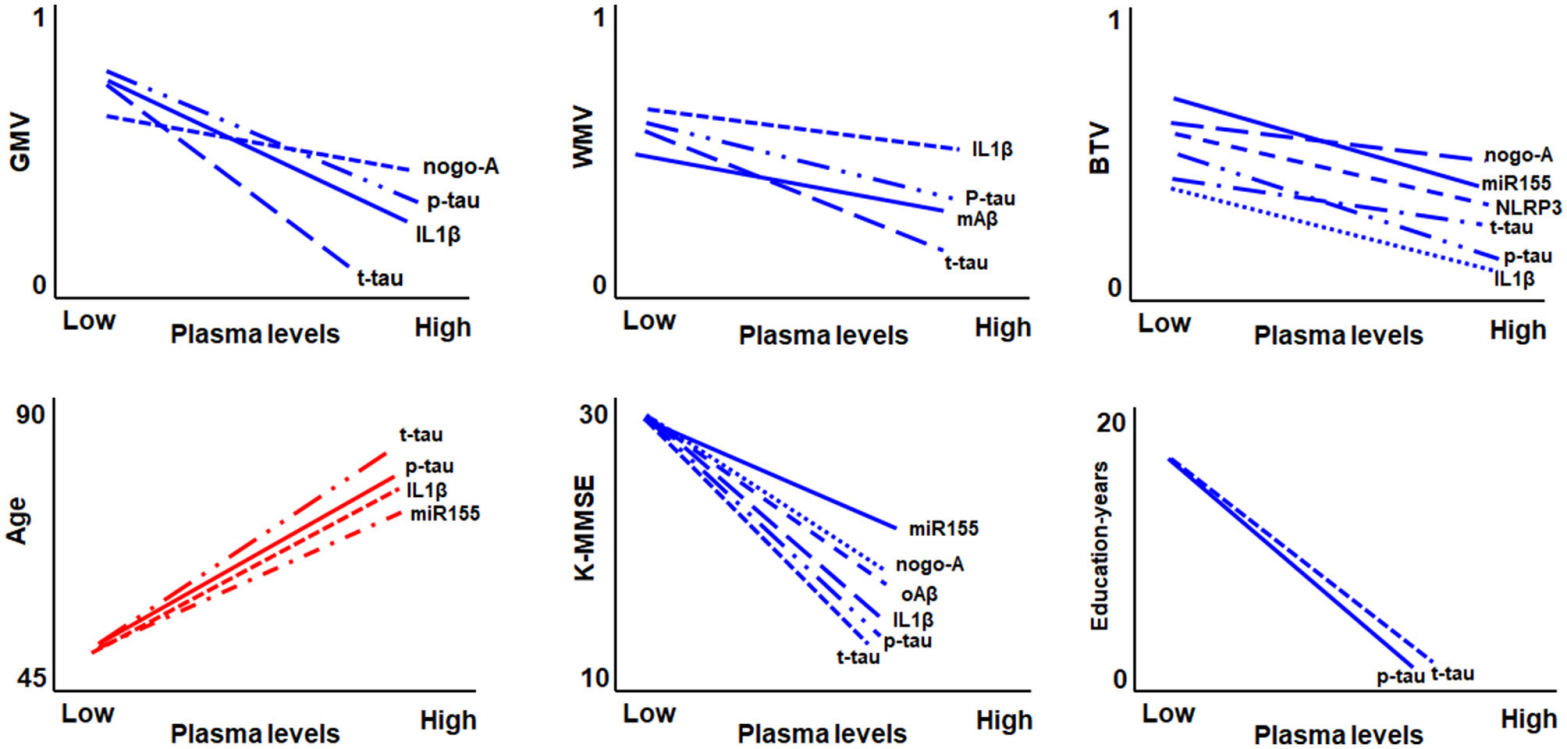
Graphical summary of the correlation between the measures of blood biomarkers and gray matter volume (GMV), white matter volume (WMV), gray-white boundary tissue volume (gwBTV), age, mini-mental state examination (K-MMSE), and education years.

Neuroinflammation, which involves the activation of the inflammatory cascade, plays a key role in the development of cognitive impairment in old age (Everett et al., 2014). However, the specific inflammatory factors contributing to brain tissue degeneration during the transition from cognitively unimpaired to impaired remain unknown (Heneka et al., 2015; Han et al., 2016). Therefore, we investigated the association between blood inflammatory biomarkers and brain tissue volume loss in elderly participants with cognitive impairment. Figure 3 shows a graphical summary of the correlation between the measures of blood biomarkers and GMV, WMV, gwBTV, age, K-MMSE, and education years. We mainly found a significant association between IL1β, or T-tau, which is a battery of blood inflammatory biomarkers, and the loss of three tissue volumes (GMW, WMV, and gwBTV). Moreover, the NLRP3 level was correlated with and associated only with gwBTV. Therefore, we will focus on IL1β, T-tau, and NLRP3 as blood inflammatory biomarkers in the discussion section.

### Interleukin 1 beta was negatively correlated and associated with GMV

IL1β levels increased significantly with age in our study. Moreover, the level of IL1β showed an increasing trend with an increase in disease severity (*r* = −0.608, *p* < 0.001) (Table 1). Previous studies indicated that higher expression of age-related proinflammatory cytokines impaired neuroplasticity and cognition (Golia et al., 2019). Neuroinflammation is characterized by activated microglia and the release of potent inflammatory mediators into the microenvironment, which can also promote the deposition of Aβ (Solfrizzi et al., 2006; Shaftel et al., 2007). IL1β is a primary signal of cerebral neuroinflammation in ongoing brain neurodegeneration. Previous reports found a negative correlation between IL1β levels and cognitive impairment levels, indicating that activated microglia and complement systems, along with increased formation of cytokines and chemokines, were involved in AD progression (Yasojima et al., 1999; Cagnin et al., 2001; Yasutake et al., 2006; Bonotis et al., 2008; Swardfager et al., 2010; Italiani et al., 2018; Baik et al., 2019). Furthermore, our results indicated a negative correlation between IL1β and GMV in the hippocampus and parahippocampus (Figure 2), which is in line with a previous report that IL1β could affect the neurons in the hippocampus (Schneider et al., 1998). This indicates that IL1β expression might impact brain morphology through processes related to neurodegeneration and might predict a decrease in the hippocampal volume in patients with cognitive decline (Frodl et al., 2012). We also found a negative correlation between IL1β and WMV in the hippocampus (Figure 2) and a negative association between IL1β and WMV in the middle temporal gyrus and the middle frontal gyrus (Table 2). White matter hyperintensities (WMH), which are related to a synergy between IL1β and injury response, could be induced by endothelial cell activation, inflammation, and ischemic injury of the brain, as well as aging and dementia (Origlia et al., 2014; Swardfager et al., 2017).

### NLRP3 level was only negatively correlated and associated with gwBTV

The NLRP3 level was negatively correlated and associated with gwBTV in the precuneus, middle temporal gyrus, and middle occipital gyrus (Table 2; Figure 2). A critical contributor to cognitive decline progression is neuroinflammation, which is driven and triggered by NLRP3 inflammasome activation (Halle et al., 2008; Ising et al., 2019). NLRP3 activated caspase-1, which drives the release of proinflammatory cytokines by microglia, disrupting neuronal connections and inducing synaptic degeneration (Heneka et al., 2013; Moraes et al., 2023). The gray-white boundary is characterized by vulnerability and sensitivity to injury (Alisafaei et al., 2020), which may explain why NLRP3 was associated only with gwBTV, rather than gray matter or white matter independently, in the early stage of AD. Caspase-1 activation and proinflammatory cytokine release preceded the alteration of AD pathology and cognitive impairment, which might suggest that NLRP3 activation occurred in the early stage of AD (Tarkowski et al., 2003; de Calignon et al., 2010).

#### Total tau pathology was negatively correlated with three tissue volumes and strongly associated with three tissue volumes

A significant decrease in the level of T-tau with increased age was also observed (Table 1). Previous studies suggested that reduced age-related autophagy function might result in aggravation of the accumulation of tau protein (Chatterjee et al., 2023), thereby accelerating the accumulation of neurofibrillary tangles (NFT). NFT counts and density in cognitively impaired elderly individuals were more numerous in the hippocampus and entorhinal compared to normal individuals. Our results showed that three tissue volumes in the hippocampus, and both GMV and WMV in most of the ROI regions, were strongly negatively correlated with T-tau (Figure 2). T-tau showed a distinct association with three brain tissue volumes (Table 2) among all blood biomarkers. Cortical neuronal loss elevates T-tau levels in AD. Changes in the brain microstructure of the cortex and white matter would be induced by axonal degeneration and neuron apoptosis based on neuroinflammation, even in the early stages of AD (Bendlin et al., 2012; Yanamandra et al., 2015). A previous study found higher CSF tau levels in the preclinical stage of AD patients, but the levels decreased as the disease progressed despite the accrual of neurofibrillary tangles (Fagan et al., 2014). At the same time, the tissue atrophy rate displayed a sigmoid curve with the MMSE score (Sabuncu et al., 2011). Decreased tau secretion and/or release, possibly resulting from the assembly of tau into larger and more stable filaments within the neurons and the loss of synapses and the neurons, might explain these observations. AD was predicted more accurately by plasma P-tau than CSF P-tau, Aβ42/Aβ40, or neurofilament light protein, as shown by a previous study (Palmqvist et al., 2021). However, the relationship between P-tau and three tissue volumes was hardly ever found (Figure 1) in this study when the impact of all blood biomarkers was considered. This may suggest that a dominant role in the tau pathology of AD might be played by T-tau.

#### Lack of correlation between oAβ and brain tissue volume loss

The absence of a significant correlation between oAβ and brain tissue volume loss (encompassing GMV, WMV, and gwBTV) is not entirely understood and may be attributed to several factors. One possibility is that the impact of oAβ on brain tissue volume might vary across different stages of AD (Wang et al., 2024). oAβ is thought to primarily influence the early stages of AD pathology, contributing to synaptic dysfunction that precedes the typical deposition of fibrillar amyloid plaques and neuronal loss (Ding et al., 2019). Studies indicate that oAβ levels are lower in the later stages of AD compared to MCI or mild dementia stages, suggesting a progression in amyloidosis that eventually plateaus (Jack et al., 2013; Youn et al., 2020). Thus, the cognitive decline observed in the advanced stages of AD may be more closely related to other pathologies, such as tau-related inflammation (Chen and Yu, 2023). The methods employed to measure oAβ and brain volumes might also influence the observed correlations. For instance, differences in biomarker detection methods, such as using plasma measurements instead of cerebrospinal fluid (CSF), could significantly affect correlations (Mroczko et al., 2018). Despite its known neurotoxic effects, the direct impact of oAβ on brain tissue volume may be less straightforward. Interactions with other pathological processes like inflammation or tau pathology may mediate oAβ's relationship with tissue volume loss (Gong et al., 2003). Additionally, compensatory brain mechanisms such as synaptic plasticity or neurogenesis could obscure the direct effects of oAβ on brain tissue volumes (Sciaccaluga et al., 2021). Not all oAβ species contribute equally to tissue volume loss; certain oligomers may exert more neurotoxic effects than others, and the specific types present in the study participants could have influenced the outcomes (Bernabeu-Zornoza et al., 2021).

Furthermore, there might be a threshold level of oAβ necessary to observe an effect on brain tissue volumes. If the levels in the study participants were below this threshold, this could account for the absence of a significant correlation (Wang et al., 2024). Finally, the sample size and the statistical methods utilized could also impact the ability to detect significant correlations. If the study was underpowered, it might fail to detect existing correlations (Mroczko et al., 2018). Further research is essential to elucidate these findings and verify the potential relationships between oAβ and brain tissue volume loss across different stages of Alzheimer's disease.

#### Positive correlation between miR155 and gwBTV

The observed positive correlation between miR155 and gwBTV in the middle temporal gyrus is likely underpinned by the multifunctional roles of miR155 within CNS, especially pertaining to neuroinflammation and neurodegenerative diseases. This correlation may stem from miR155′s involvement in modulating neuroinflammation, affecting CNS cell survival and repair mechanisms, influencing synaptic plasticity, and its association with the pathologies of neurodegenerative disorders. miR155 is recognized as a crucial regulator of neuroinflammation, impacting the behavior of microglia, the primary immune cells in the brain. An upregulation of miR155 tends to promote a proinflammatory state in microglia, a common occurrence in several neurodegenerative diseases, including AD (Zingale et al., 2022). Beyond its inflammatory roles, miR155 also impacts CNS cell survival and repair by influencing gene expression related to cell survival, apoptosis, and neurogenesis. These processes are vital for maintaining the integrity of the gray-white matter boundary, potentially accounting for the positive correlation observed in the middle temporal gyrus (Alivernini et al., 2018). The middle temporal gyrus plays a key role in cognitive functions, including language processing and semantic memory. miR155′s influence on synaptic plasticity, crucial for learning and memory, may contribute to maintaining or increasing tissue volume in this area (Zingale et al., 2022). Furthermore, miR155′s regulatory effects on neuroinflammation and its interactions with pathological markers such as tau and amyloid beta could indirectly influence brain tissue volumes.

Moreover, the middle temporal gyrus is among the regions impacted early in the progression of AD, and its involvement in AD pathology may be linked to volumetric changes noted in this area (Alivernini et al., 2018). Changes in the brain structure related to cognitive impairments associated with the temporal lobe often involve structural abnormalities in the temporal cortex, including localized cortical thickening and a blurring of the gray-white matter boundary. These changes are due to aberrations such as abnormal neurons and focal reductions in myelinated fibers, which lead to reduced signal contrasts between gray and white matter (Blackmon et al., 2019). Factors like gray matter atrophy would increase signal intensity values due to decreased water content, while demyelination processes would decrease white matter intensity due to increased water content (Uribe et al., 2018). Despite these insights, further research is crucial to deepening our understanding of how miR155′s modulation affects brain tissue volume changes, particularly in participants experiencing cognitive decline.

### Limitation

Several limitations were present in this study. First, the sample size was small compared to other studies on plasma biomarkers. Therefore, larger sample sizes are required for future studies. Second, some complications, such as a lack of physical exercise and different food habits, which may be experienced by older people, were not evaluated and normalized in this study. Therefore, more attention should be paid to controlling the above-mentioned factors in future studies. Finally, the current study estimated cross-sectional data, so the longitudinal changes in the levels of biomarkers need to be investigated in future studies. Longitudinal evaluation of biomarkers may reveal discrepancies between time points and lead to the discovery of more prominent biomarkers.

## Conclusions

Our study explored the relationship between inflammatory biomarkers and brain tissue volume loss in older individuals with cognitive impairment. We found that IL1β, P-tau, and T-tau levels increased with age and were strongly correlated with the severity of cognitive deficits. Notably, these biomarkers showed a significant negative correlation with brain tissue volumes, specifically GMV, WMV, and gwBTV. The hippocampus, a region pivotal for memory and cognitive function, demonstrated a particularly pronounced negative association with IL1β and T-tau levels, highlighting the link between inflammation, neurodegeneration, and cognitive decline. Additionally, NLRP3 levels were negatively correlated with gwBTV, further substantiating the connection between inflammatory processes and brain structure deterioration in cognitive impairment. These findings highlight the potential of inflammatory mediators as biomarkers for detecting and monitoring neurodegeneration in the aging brain. While our results are compelling, they pave the way for future research to solidify our understanding of how neuroinflammation contributes to the progression of cognitive impairment in older individuals. Such studies are essential for developing targeted interventions to mitigate the impact of these debilitating conditions.

## Data availability statement

The raw data supporting the conclusions of this article will be made available by the authors, without undue reservation.

## Ethics statement

The studies involving humans were approved by the Institutional Review Board (IRB) of Kyung Hee University Hospital at Gangdong (KHNMC IRB 2009-056, KHNMC GRRB 2009-004, KHNMC IRB 2011-059, and KHNMC GRRB 2011-008). The studies were conducted in accordance with the local legislation and institutional requirements. The participants provided their written informed consent to participate in this study.

## Author contributions

KL: Data curation, Investigation, Methodology, Writing – original draft, Writing – review & editing. SK: Conceptualization, Data curation, Formal analysis, Investigation, Methodology, Resources, Supervision, Validation, Visualization, Writing – original draft, Writing – review & editing. YT: Data curation, Formal analysis, Methodology, Software, Writing – original draft, Writing – review & editing. SJ: Formal analysis, Methodology, Software, Writing – review & editing. YC: Formal analysis, Methodology, Software, Writing – review & editing. HR: Data curation, Investigation, Writing – review & editing. SP: Data curation, Investigation, Writing – review & editing. C-WR: Data curation, Investigation, Writing – review & editing. W-IL: Methodology, Resources, Writing – review & editing. EK: Data curation, Investigation, Writing – review & editing. G-HJ: Conceptualization, Data curation, Formal analysis, Funding acquisition, Methodology, Project administration, Software, Supervision, Validation, Visualization, Writing – original draft, Writing – review & editing.
