# Supplementary material for: Correlation of brain tissue volume loss with inflammatory biomarkers IL1β, P-tau, T-tau, and NLPR3 in the aging cognitively impaired population
Source: Front Aging Neurosci. 2024 Jul 23;16:1388654. doi: 10.3389/fnagi.2024.1388654 (PMC11300291; doi:10.3389/fnagi.2024.1388654)
Supplement: Supplementary file 1 [file Table_1.pdf]

Correlation of brain tissue volume loss with inflammatory biomarkers IL1b, P-tau, T-tau, and NLPR3 in the aging cognitively impaired population

Supplementary Results

Table S1. Significantly associated anatomical locations of results of voxel-based multiple regression analyses between gray matter volume (GMV) and levels of blood-based biomarkers of all participants after being adjusted for participants' age and education-year

Group analysis	Cluster size	Cluster location	BA	Talairach coordinates	Z score	ROI
**(-) IL1β**						
	88119	Lt Limbic Parahippocampal Gyrus	35	-24.39, -21.91, -19.83	6.027	ROI
		Lt Lentiform Nucleus Medial Globus Pallidus		-18.94, -7.61, -7.57	5.926	
		Lt Limbic Uncus	34	-14.59, 3.32, -18.63	5.773	
	3265	Lt Middle Occipital Gyrus	19	-30.7, -74.1, 23.76	4.519	ROI
		Lt Parietal Precuneus	19	-28.14, -82.54, 37.87	3.892	ROI
	989	Rt Frontal Precentral Gyrus	4	54.05, -17.77, 35.94	4.166	
		Rt Parietal Postcentral Gyrus	40	58.38, -26.13, 20.36	3.163	
	3029	Rt Middle Frontal Gyrus	10	28.17, 47.74, 16.03	4.005	ROI
		Rt Limbic Anterior Cingulate	32	10.38, 44.2, -4.87	3.604	
	952	Lt Limbic Anterior Cingulate	32	-18.89, 44.83, 4.15	3.707	
		Lt Superior Frontal Gyrus	10	-20.49, 46.07, 20.46	2.928	
	1517	Lt Posterior Inferior Semi-Lunar Loblue		-36.85, -66.79, -36.46	3.654	
**GMV**		Lt Posterior Cerebeller Tonsil		-39.6, -58.39, -35.71	3.629	
	1073	Rt Inferior Parietal Lobule	40	53.86, -50.69, 40.92	3.562	
		Rt Superior Temporal Gyrus	39	47.17, -58.35, 18.47	2.995	
	314	Rt Middle Frontal Gyrus	6	33.09, -2.2, 50.57	3.484	ROI
	259	Lt Inferior Parietal Lobule	40	-37.57, -36.46, 28.56	3.410	
		Lt Parietal Postcentral Gyrus	2	-33.42, -31.16, 31.84	2.478	
	469	Lt Middle Occipital Gyrus	19	-40.21, -80.73, 5.41	3.341	ROI
		Lt Middle Temporal Gyrus	39	-48.65, -74.62, 15.3	2.691	ROI
	511	Lt Middle Frontal Gyrus	6	-33.38, 11.64, 41.3	3.338	ROI
	473	L Middle Occipital Gyrus	18	-15.37, -94.22, 12.66	3.287	ROI
		Lt Occipital Cuneus	17	-6.93, -93.48, 4.76	2.591	
	254	Lt Superior Temporal Gyrus	42	-52.65, -32.28, 15.19	3.285	
**(-) p-tau**						
	497	Lt Parietal Cingulate Gyrus	31	-8.53. -45.65. 34.94	4.580	
**GMV**		Lt Limbic Cingulate Gyrus	31	-16.79, -39.63, 31.32	4.468	
	155	Lt Occipital Cuneus	18	-20.94, -80.16, 17.91	4.263	
**(-) t-tau**						
	242269	Rt Limbic Parahippocampal Gyrus		26.9, -6.19, -9.37	7.277	ROI
		Lt Lentifrom Nucleus Medial Globus Pallidus Amygdala		-18.95, -10.41, -7.84	7.184	
**GMV**		Lt Limbic Parahippocampal Gyrus		-29.98, -0.18, -11.11	7.165	ROI
		Rt Posterior Cerebeller Tonsil Amygdala		7.75, -39.68, -41.24	2.930	
	933	Rt Occipital Cuneus	18	19.35, -95.67, 11.76	3.029	

		Rt Middle Occipital Gyrus	18	17.91, -89.2, 17.75	2.949	ROI
		Rt Occipital Cuneus	17	12.45, -80.26, 13.1	2.626	
	364	Lt Posterior Cerebellar Tonsil		-10.44, -55.74, -34.96	3.024	
		Lt Anterior Nodule		-10.48, -49.15, -30.28	2.720	
	101	Rt Middle Frontal Gyrus	6	27.38, -10.2, 60.53	2.627	ROI
	173	Rt Middle Frontal Gyrus	8	27.83, 15.03, 33.2	2.136	ROI

BA - Brodmann area

The anatomical locations are corresponding to the Figure 2 which shows the result of voxel-based multiple regression analyses between GMV of all participants and levels of blood-based biomarkers. Monomer Aβ (mAβ), NLRP3, miR155, oligomer Aβ (oAβ), and Nogo-A were not significant. There were no positive relationships between GMV and levels of blood-based biomarkers.

Table S2. Significantly associated anatomical locations of results of voxel-based multiple regression analyses between white matter volume (WMV) and levels of blood-based biomarkers of all participants after being adjusted for participants' age and education-year

Group analysis	Cluster size	Cluster location	BA	Talairach coordinates	Z score	ROI
(-) t-tau						
WMV	5118	Lt Limbic Parahippocampal Gyrus		-28.41, -5.74, -26.47	5.350	ROI
		Lt Inferior Frontal Gyrus	47	26.97, -20.68, -22.6	4.166	
	4855	Rt Limbic Parahippocampal Gyrus		25.61, -15.05, -19.69	4.818	ROI
		Rt Limbic Uncus Amygdala		25.9, -4.88, -22.78	4.808	
		Rt Frontal Sub-Gyral		29.62, 30.35, 6.3	4.380	
	738	Lt Middle Temporal Gyrus	21	-63.49, -23.41, -3.07	4.799	ROI
	544	Rt Parietal Sub-Gyral		40.08, -38.92, 36.4	4.001	
	450	Rt Limbic Cingulate	32	8.4, 16.53, 33.01	3.994	
	840	Lt Parietal Precuneus		-14.18, -43.61, 43.15	3.973	ROI
	1041	Rt Parietal Precuneus	7	14.85, -58.39, 48.99	3.905	ROI
		Rt Limbic Cingulate gyrus		12.28, -39.03, 38.62	3.564	
	897	Lt Inferior Frontal Gyrus		-37, 34.88, 5.6	3.809	
	871	Lt Middle Temporal Gyrus		-37.66, -74.19, 24.99	3.664	ROI
		Lt Parietal Supramarginal Gyrus		-39.13, -51.48, 37.93	3.436	
	185	Lt Posterior Cerebellar Tonsil		-42.3, -49.6, -38.97	3.580	
	199	Rt Temporal Sub-Gyral		50.61, -10.99, -18.88	3.546	
	160	Rt Medial Frontal Gyrus	25	6.27, 11.73, -18.83	3.540	ROI
	108	Rt Limbic Anterior Cingulate	32	6.13, 46.36, 2.02	3.505	
	183	Rt Sub-Lobar Extra-Nuclear		12.86, 5.02, 6.32	3.292	
	143	Lt Limbic Cingulate Gyrus		-11.19, -42.06, 27.13	2.244	

BA - Brodmann area

The anatomical locations are corresponding to the Figure 2 which shows the result of voxel-based multiple regression analyses between WMV of all participants and levels of blood-based biomarkers. Monomer Aβ (mAβ), NLRP3, miR155, oligomer Aβ (oAβ), Nogo-A, and P-tau were not significant. There were no positive relationships between WMV and levels of blood-based biomarkers.

Table S3 Significantly associated anatomical locations of results of voxel-based multiple regression analyses between gray-white matter boundary tissue volume (gwBTV) and levels of blood-based biomarkers of all participants after being adjusted for participants' age and education-year

Group analysis	Cluster size	Cluster location	BA	Talairach coordinates	Z score	ROI
**(-) IL1β**						
	2642	Lt Sub-lobar Extra-Nuclear WM		-21.52, -9.98, 11.98	5.09	
		Lt Sub-lobar Extra-Nuclear WM		-3.92, -8.13, 11.55	3.95	
		Lt Sub-lobar Thalamus GM Medial Dorsal Nucleus		-5.72, -17.84, 5.19	3.88	
	1482	Rt Sub-lobar Extra-Nuclear WM		35.23, -3.53, -8.07	4.86	
		Rt Limbic Parahippocampal Gyrus WM		29.69, -9.76, -11.46	4.06	ROI
		Rt Temporal Sub-Gyral WM		25.94, -18.39, -9.64	3.71	
	786	Inter-Hemispheric		0.73, -6.2, 10.91	4.36	
		Inter-Hemispheric		-0.26, -17.64, 12.51	3.83	
	306	Rt Medial Frontal Gyrus WM		14.89, 58.88, 8.31	4.28	
	124	Lt Middle Temporal Gyrus WM		-60.25, -27.11, -3.81	4.06	ROI
gwBTV	455	Lt Sub-lobar Extra-Nuclear WM		-30.58, -23.19, -6.54	4.02	
		Lt Sub-lobar Lateral Ventricle CSF		-27.74, -17.18, -10.43	3.82	
	189	Lt Occipital Lingual Gyrus WM		-9.49, -87.75, -10.5	3.97	
		Lt Occipital Lingual Gyrus GM	17	-14.18, -94.6, -7.63	3.72	
	126	Lt Middle Frontal Gyrus GM	46	-42.65, 38.74, 14.43	3.94	ROI
	175	Left Brainstem Midbrain		-14.76, -10.72, -9.6	3.93	
		Lt Limbic Parahippocampal Gyrus		-12.82, -6.4, -15.46	3.69	ROI
	302	Rt Sub-lobar Extra-Nuclear WM		2.82, -6.24, -7.98	3.92	
		Rt Sub-lobar Extra-Nuclear WM		3.66, -2.29, -0.38	3.79	
	241	Rt Limbic Cingulate Gyrus		2.41, 17.2, 31.17	3.92	
		Inter-Hemispheric		1.65, 27.57, 21.33	3.5	
	105	Rt Superior Frontal Gyrus WM		19.17, 50.83, 32.84	3.91	
	239	Lt Sub-lobar Extra-Nuclear WM		-36.94, -3.07, -10.15	3.83	
	128	Rt Limbic Parahippocampal Gyrus WM		27.13, -4.54, -26.32	3.83	ROI
	126	Lt Superior Temporal Gyrus WM		-25.52, 4.73, -32.64	3.64	
	469	Rt Sub-lobar Extra-Nuclear WM		20.96, -13.61, 18.66	3.62	
		Rt Sub-lobar Extra-Nuclear WM		18.29, -6.46, 12.98	3.48	
**(-) t-tau**						
	2050	Rt Sub-lobar Extra-Nuclear WM		30.61, -9.85, -10.55	5	
		Rt Sub-lobar Extra-Nuclear WM		34.31, -3.44, -8.98	4.84	
		Rt Sub-lobar Lateral Ventricle CSF		26.87, -16.53, -9.45	4.19	
	2647	Lt Sub-lobar Extra-Nuclear WM		-20.59, -9.99, 11.99	4.89	
		Rt Sub-lobar Extra-Nuclear WM		3.66, -2.29, -0.38	4.27	
		Lt Sub-lobar Thalamus GM		-3.89, -7.95, 9.76	4.12	
	672	Inter-Hemispheric		-0.19, -6.11, 10	4.49	
		Rt Sub-lobar Thalamus GM		9.88, -9.74, 17.94	3.46	
	240	Lt Middle Temporal Gyrus WM		-60.25, -26.18, -3.73	4.46	ROI
	481	Rt Superior Frontal Gyrus WM		15.82, 58.88, 8.32	4.44	
	546	Rt Limbic Cingulate Gyrus		2.42, 18.22, 30.37	4.3	
	151	Lt Limbic Parahippocampal Gyrus GM	30	-13.12, -38.79, -1.42	4.29	ROI
gwBTV		Lt Limbic Parahippocampal Gyrus GM	28	-18.56, -29.68, -7.86	3.74	ROI
	736	Lt Temporal Sub-Gyral GM Hippocampus		-30.57, -23.1, -7.44	4.28	
	544	Lt Temporal Sub-Gyral WM		-36.96, -4.09, -9.35	4.27	
	220	Rt Superior Frontal Gyrus WM		17.32, 49.91, 32.72	4.24	
	393	Lt Frontal Precentral Gyrus WM		-54.14, -9.7, 29.47	4.2	
	168	Lt Limbic Parahippocampal Gyrus		-14.74, -10.55, -11.39	3.99	ROI
	249	Rt Inferior Frontal Gyrus WM		32.53, 25.36, -5.38	3.9	
	124	Inter-Hemispheric Cingulate Gyrus		1.24, -4.68, 43.49	3.9	
	361	Lt Limbic Anterior Cingulate		-2.69, 29.77, -1.06	3.87	
		Inter-Hemispheric		0.16.28, 2.22	3.79	
	205	Lt Frontal Precentral Gyrus GM	4	-44.13, -16.47, 40.71	3.78	
	111	Rt Medial Frontal Gyrus WM		7.1, 40.56, 32.56	3.62	
	108	Rt Medial Frontal Gyrus WM		12.29, -4.24, 48.22	3.55	
		Rt Medial Frontal Gyrus WM		9.43, -15.76, 50.69	3.54	

BA - Brodmann area

The anatomical locations are corresponding to the Figure 2 which shows the result of voxel-based multiple regression analyses between gwBTV of all participants and levels of blood-based biomarkers. Monomer Aβ (mAβ), NLRP3, miR155, oligomer Aβ (oA β), Nogo-A, and P-tau were not significant. There were no positive relationships between gwBTV and levels of blood-based biomarkers.

Table S4 Results of Pearson correlation analysis of brain tissue volumes with blood biomarkers in specific brain areas related to cognitive function.

Index	Tissue volume	mAβ	oligomer	IL1β	miR155	NLRP3	nogo A	p-tau	t-tau
Anterior cingulate	GMV	r= -0.034<br>P= 0.721	r= -0.150<br>P= 0.116	r= -0.297<br>P= 0.002	r= -0.194<br>P= 0.042	r= -0.025<br>P= 0.796	r= -0.190<br>P= 0.046	r= -0.357<br>P= 0.0001	r= -0.336<br>P= 0.0003
	WMV	r= -0.072<br>P= 0.453	r= -0.157<br>P= 0.099	r= -0.278<br>P= 0.003	r= -0.393<br>P< 0.0001	r= -0.059<br>P= 0.537	r= -0.388<br>P< 0.0001	r= -0.281<br>P= 0.003	r= -0.241<br>P= 0.011
	gwBTV	r= -0.083<br>P= 0.384	r= -0.216<br>P= 0.023	r= -0.346<br>P= 0.0002	r= -0.257<br>P= 0.007	r= 0.029<br>P= 0.766	r= -0.178<br>P= 0.062	r= -0.402<br>P< 0.0001	r= -0.423<br>P< 0.0001
Brain stem	GMV	r= 0.034<br>P=0.724	r= 0.026<br>P= 0.786	r= 0.0116<br>P= 0.904	r= 0.067<br>P= 0.486	r= -0.056<br>P= 0.561	r= 0.052<br>P= 0.591	r= 0.018<br>P= 0.853	r= -0.029<br>P= 0.762
	WMV	r= 0.014<br>P= 0.886	r= -0.205<br>P= 0.031	r= -0.335<br>P= 0.0003	r= -0.380<br>P< 0.0001	r= 0.004<br>P= 0.970	r= -0.230<br>P= 0.015	r= -0.277<br>P= 0.003	r= -0.272<br>P= 0.004
	gwBTV	r= 0.129<br>P= 0.177	r= -0.190<br>P= 0.046	r= -0.335<br>P= 0.0003	r= -0.248<br>P= 0.009	r= 0.144<br>P= 0.133	r= -0.172<br>P= 0.071	r= -0.357<br>P= 0.0001	r= -0.329<br>P= 0.0004
Cerebellum	GMV	r= 0.061<br>P= 0.528	r= -0.048<br>P= 0.615	r= -0.217<br>P= 0.022	r= -0.164<br>P= 0.086	r= 0.059<br>P= 0.541	r= -0.079<br>P= 0.412	r= -0.183<br>P= 0.054	r= -0.210<br>P= 0.027
	WMV	r= -0.036<br>P= 0.709	r= -0.249<br>P= 0.008	r= -0.265<br>P= 0.005	r= -0.379<br>P< 0.0001	r= -0.095<br>P= 0.321	r= -0.252<br>P= 0.008	r= -0.217<br>P= 0.023	r= -0.197<br>P= 0.038
	gwBTV	r= 0.058<br>P= 0.543	r= 0.001<br>P= 0.990	r= -0.275<br>P= 0.004	r= -0.254<br>P= 0.007	r= 0.149<br>P= 0.118	r= -0.087<br>P= 0.365	r= -0.249<br>P= 0.008	r= -0.294<br>P= 0.002
Pons	GMV	r= 0.015<br>P= 0.874	r= 0.113<br>P= 0.239	r= 0.165<br>P= 0.083	r= 0.162<br>P= 0.090	r= -0.033<br>P= 0.729	r= 0.157<br>P= 0.100	r= 0.141<br>P= 0.141	r= 0.129<br>P=0.176
	WMV	r= 0.050<br>P= 0.603	r= -0.155<br>P= 0.104	r= -0.275<br>P= 0.004	r= -0.310<br>P= 0.0009	r= 0.040<br>P= 0.673	r= -0.187<br>P= 0.049	r= -0.203<br>P= 0.033	r= -0.210<br>P= 0.027
	gwBTV	r= 0.185<br>P= 0.052	r= -0.040<br>P= 0.678	r= -0.137<br>P= 0.151	r= -0.046<br>P= 0.631	r= 0.181<br>P= 0.058	r= -0.088<br>P= 0.361	r= -0.124<br>P= 0.195	r= -0.100<br>P= 0.295
Striatum	GMV	r= 0.083<br>P= 0.388	r= -0.166<br>P= 0.082	r= -0.305<br>P= 0.001	r= -0.316<br>P= 0.0007	r= -0.057<br>P= 0.553	r= -0.112<br>P= 0.241	r= -0.304<br>P= 0.001	r= -0.257<br>P= 0.006
	WMV	r= -0.204<br>P= 0.032	r= 0.003<br>P= 0.976	r= -0.105<br>P= 0.272	r= -0.028<br>P= 0.768	r= 0.055<br>P= 0.564	r= -0.065<br>P= 0.499	r= -0.132<br>P= 0.169	r= -0.174<br>P= 0.067
	gwBTV	r= -0.097<br>P= 0.314	r= -0.177<br>P= 0.064	r= -0.367<br>P= 0.0001	r= -0.308<br>P= 0.001	r= 0.080<br>P= 0.401	r= -0.164<br>P= 0.085	r= -0.379<br>P< 0.0001	r= -0.355<br>P= 0.0001
Caudate nucleus	GMV	r= -0.029<br>P= 0.761	r= -0.138<br>P= 0.149	r= -0.374<br>P= 0.0001	r= -0.329<br>P= 0.0004	r= 0.026<br>P= 0.789	r= -0.184<br>P= 0.054	r= -0.386<br>P< 0.0001	r= -0.369<br>P= 0.0001
	WMV	r= -0.068<br>P= 0.476	r= -0.064<br>P= 0.502	r= -0.250<br>P= 0.008	r= -0.260<br>P= 0.006	r= 0.051<br>P= 0.592	r= -0.130<br>P= 0.174	r= -0.262<br>P= 0.006	r= -0.280<br>P= 0.003
	gwBTV	r= -0.079<br>P= 0.408	r= -0.175<br>P= 0.066	r= -0.371<br>P= 0.0001	r= -0.358<br>P= 0.0001	r= 0.070<br>P= 0.467	r= -0.203<br>P= 0.033	r= -0.388<br>P< 0.0001	r= -0.366<br>P= 0.0001
Entorhinal cortex	GMV	r= -0.080<br>P= 0.403	r= -0.164<br>P= 0.085	r= -0.457<br>P< 0.0001	r= -0.320<br>P= 0.0006	r= 0.104<br>P= 0.276	r= -0.302<br>P= 0.001	r= -0.450<br>P< 0.0001	r= -0.511<br>P< 0.0001
	WMV	r= -0.131<br>P= 0.172	r= -0.219<br>P= 0.021	r= -0.378<br>P< 0.0001	r= -0.324<br>P= 0.0005	r= 0.094<br>P= 0.329	r= -0.206<br>P= 0.031	r= -0.354<br>P= 0.0001	r= -0.377<br>P< 0.0001
	gwBTV	r= -0.055<br>P= 0.563	r= -0.146<br>P= 0.127	r= -0.385<br>P< 0.0001	r= -0.360<br>P= 0.0001	r= 0.111<br>P= 0.246	r= -0.284<br>P= 0.003	r= -0.422<br>P< 0.0001	r= -0.417<br>P< 0.0001
Thalamus	GMV	r= 0.082<br>P= 0.391	r= -0.157<br>P= 0.101	r= -0.256<br>P= 0.006	r= -0.251<br>P= 0.008	r= 0.014<br>P= 0.883	r= -0.127<br>P= 0.184	r= -0.290<br>P= 0.002	r= -0.303<br>P= 0.001
	WMV	r= -0.105<br>P= 0.271	r= -0.017<br>P= 0.858	r= -0.225<br>P= 0.017	r= -0.152<br>P= 0.111	r= 0.043<br>P= 0.654	r= -0.078<br>P= 0.418	r= -0.192<br>P= 0.044	r= -0.181<br>P= 0.057
	gwBTV	r= 0.015<br>P= 0.876	r= -0.201<br>P= 0.034	r= -0.366<br>P= 0.0001	r= -0.352<br>P= 0.0002	r= 0.037<br>P= 0.703	r= -0.161<br>P= 0.092	r= -0.376<br>P< 0.0001	r= -0.369<br>P= 0.0001
Inferior frontal gyrus	GMV	r= -0.103<br>P= 0.284	r= -0.100<br>P= 0.298	r= -0.296<br>P= 0.002	r= -0.230<br>P= 0.015	r= -0.021<br>P= 0.828	r= -0.243<br>P= 0.010	r= -0.327<br>P= 0.0005	r= -0.306<br>P= 0.001
	WMV	r= -0.128<br>P= 0.181	r= -0.161<br>P= 0.091	r= -0.336<br>P= 0.0003	r= -0.318<br>P= 0.0007	r= -0.034<br>P= 0.723	r= -0.286<br>P= 0.002	r= -0.372<br>P= 0.0001	r= -0.317<br>P= 0.0007
	gwBTV	r= -0.185<br>P= 0.052	r= -0.112<br>P= 0.240	r= -0.314<br>P= 0.0008	r= -0.186<br>P= 0.051	r= -0.634<br>P= 0.506	r= -0.289<br>P= 0.002	r= -0.348<br>P= 0.0002	r= -0.352<br>P= 0.0002

**Superior frontal gyrus**	GMV	r= -0.045<br>P= 0.643	r= -0.108<br>P= 0.258	r= -0.278<br>P= 0.003	r= -0.170<br>P= 0.074	r= -0.075<br>P= 0.433	r= -0.270<br>P= 0.004	r= -0.330<br>P= 0.0004	r= -0.282<br>P= 0.003
	WMV	r= -0.074<br>P= 0.442	r= -0.112<br>P= 0.240	r= -0.269<br>P= 0.004	r= -0.237<br>P= 0.012	r= 0.077<br>P= 0.423	r= -0.135<br>P= 0.158	r= -0.239<br>P= 0.002	r= -0.290<br>P= 0.002
	gwBTV	r= -0.143<br>P= 0.134	r= -0.080<br>P= 0.407	r= -0.256<br>P= 0.007	r= -0.020<br>P= 0.833	r= 0.009<br>P= 0.924	r= -0.096<br>P= 0.316	r= -0.281<br>P= 0.003	r= -0.269<br>P= 0.004
**Inferior temporal gyrus**	GMV	r= -0.163<br>P= 0.088	r= -0.154<br>P= 0.108	r= -0.392<br>P< 0.0001	r= -0.124<br>P= 0.196	r= -0.061<br>P= 0.523	r= -0.235<br>P= 0.013	r= -0.387<br>P< 0.0001	r= -0.425<br>P< 0.0001
	WMV	r= -0.084<br>P= 0.383	r= -0.191<br>P= 0.044	r= -0.259<br>P= 0.006	r= -0.292<br>P= 0.002	r= -0.006<br>P= 0.947	r= -0.199<br>P= 0.036	r= -0.298<br>P= 0.002	r= -0.215<br>P= 0.023
	gwBTV	r= -0.121<br>P= 0.205	r= 0.122<br>P= 0.204	r= -0.064<br>P= 0.506	r= 0.227<br>P= 0.017	r= -0.088<br>P= 0.360	r= 0.097<br>P= 0.312	r= -0.029<br>P= 0.762	r= -0.092<br>P= 0.335
**Superior temporal gyrus**	GMV	r= -0.058<br>P= 0.545	r= -0.129<br>P= 0.178	r= -0.347<br>P= 0.0002	r= -0.225<br>P= 0.017	r= 0.083<br>P= 0.388	r= -0.224<br>P= 0.018	r= -0.395<br>P< 0.0001	r= -0.373<br>P= 0.0001
	WMV	r= -0.125<br>P= 0.192	r= -0.234<br>P= 0.014	r= -0.201<br>P= 0.035	r= -0.254<br>P= 0.007	r= -0.074<br>P= 0.442	r= -0.280<br>P= 0.003	r= -0.212<br>P= 0.025	r= -0.214<br>P= 0.024
	gwBTV	r= -0.162<br>P= 0.089	r= -0.017<br>P= 0.859	r= -0.184<br>P= 0.054	r= -0.011<br>P= 0.912	r= -0.052<br>P= 0.591	r= -0.096<br>P= 0.314	r= -0.214<br>P= 0.024	r= -0.253<br>P= 0.007

Results of Pearson correlation for all blood biomarkers are listed as coefficient (r) and p-value.

Abbreviation: GMV, gray matter volume; WMV, white matter volume; gwBTV, gray-white matter boundary tissue volume; mAβ, monomer amyloid-beta; oAβ, oligomeric amyloid-beta; IL1β, interleukin 1-beta; miR155, microRNA-155; P-tau, phosphorylated tau; and T-tau, total tau.

Supplementary Table S5 Result of power calculation based on our sample size and correlation analysis for IL1β, NLRP3, P-tau, and T-tau in the selected brain areas.

**Biomarkers**	**ROI**	**Brain tissue**	**Power**	**Beta**	**ρ1**
**IL1 β**	Hippocampus	GMV	0.995	0.005	-0.408
		WMV	0.540	0.460	-0.195
		gwBTV	0.658	0.342	-0.223
**NLRP3**	Mid OG	GMV	0.784	0.216	-0.257
	Precuneus	WMV	0.549	0.451	-0.197
	Mid TG	gwBTV	0.609	0.391	-0.211
**p-tau**	Hippocampus	GMV	0.992	0.008	-0.397
		WMV	0.527	0.473	-0.192
		gwBTV	0.770	0.230	-0.253
**T-tau**	Hippocampus	GMV	0.999	0.00005	-0.510
		WMV	0.735	0.265	-0.243
		gwBTV	0.742	0.258	-0.245

The power that is the probability of rejecting a false null hypothesis was calculated with 111 participants, alpha= 0.0, and ρ0=0, where alpha is the probability of rejecting a true null hypothesis.
Beta is the probability of accepting a false null hypothesis and ρ1 is the value of the population correlation under the alternative hypothesis.

The results show that first, IL1β of a sample size of 111 achieves 99% power to detect a difference of 0.408 between the null hypothesis correlation of 0.000 and the alternative hypothesis correlation of -0.408 using a two-sided hypothesis test with a significance level of 0.05. Second, NLRP3 of a sample size of 111 achieves 78% power to detect a difference of 0.257 between the null hypothesis correlation of 0.000 and the alternative hypothesis correlation of -0.257 using a

two-sided hypothesis test with a significance level of 0.05. Third, the P-tau of a sample size of 111 achieves 99% power to detect a difference of 0.397 between the null hypothesis correlation of 0.000 and the alternative hypothesis correlation of -0.397 using a two-sided hypothesis test with a significance level of 0.05. Finally, the T-tau of a sample size of 111 achieves 100% power to detect a difference of 0.510 between the null hypothesis correlation of 0.000 and the alternative hypothesis correlation of -0.510 using a two-sided hypothesis test with a significance level of 0.05.
